# Genetic variation in the C-type lectin receptor *CLEC4M* in type 1 von Willebrand Disease patients

**DOI:** 10.1371/journal.pone.0192024

**Published:** 2018-02-01

**Authors:** Eric Manderstedt, Christina Lind-Halldén, Stefan Lethagen, Christer Halldén

**Affiliations:** 1 Biomedicine, Kristianstad University, Kristianstad, Sweden; 2 University of Copenhagen, Copenhagen, Denmark; 3 Sobi, Stockholm, Sweden; 4 Lund University, Lund, Sweden; Institut d'Investigacions Biomediques de Barcelona, SPAIN

## Abstract

von Willebrand factor (VWF) levels in healthy individuals and in patients with type 1 von Willebrand disease (VWD) are influenced by genetic variation in several genes, e.g. *VWF*, *ABO*, *STXBP5* and *CLEC4M*. This study aims to screen comprehensively for *CLEC4M* variants and investigate their association with type 1 VWD in the Swedish population. In order to screen for *CLEC4M* variants, the *CLEC4M* gene region was re-sequenced and the polymorphic neck region was genotyped in 106 type 1 VWD patients from unrelated type 1 VWD families. Single nucleotide variants (SNV) and variable number tandem repeat (VNTR) allele and genotype frequencies were then compared with 294 individuals from the 1000Genomes project and 436 Swedish control individuals. Re-sequencing identified a total of 42 SNVs. Rare variants showed no accumulation in type 1 VWD patients and are not thought to contribute substantially to type 1 VWD. The only missense mutation (rs2277998, NP_001138379.1:p.Asp224Asn) had a higher frequency in type 1 VWD patients than in controls (4.9%). The VNTR genotypes 57 and 67 were observed at higher frequencies than expected in type 1 VWD patients (6.4% and 6.2%) and showed an increase in patients compared with controls (7.4% and 3.1%). Strong linkage disequilibrium in the *CLEC4M* region makes it difficult to distinguish between the effect of the missense mutation and the VNTR genotypes. In conclusion, heterozygous VNTR genotypes 57 and 67 of *CLEC4M* were highly enriched and are the most likely mechanism through which *CLEC4M* contributes to disease in the Swedish type 1 VWD population.

## Introduction

von Willebrand disease (VWD) is characterized by low levels of, or defective plasma von Willebrand factor (VWF) and is classified into three different types depending on the nature of the disease. Type 1 VWD is the least serious subtype accounting for approximately 70% of diagnosed cases and is defined as a partial deficiency of functionally normal VWF. In most cases it shows dominant inheritance [1]. Even in well-defined type 1 VWD patient groups approximately 35% of all type 1 VWD patients do not have a mutation in the promoter, coding sequence or splice junctions of the *VWF* gene [1]. This suggests that other genes affect the level of VWF and contribute to the disease. The most important gene identified thus far is the *ABO* blood group gene. The VWF levels of individuals with blood group O are reduced by 30% in comparison to non-O individuals and blood group O is more common in the type 1 VWD disease population in comparison with the type 2 VWD and normal populations [2]. Patients with type O-blood exhibit an increase of VWF clearance leading to a reduction of the half-life for VWF [3]. A meta-analysis of several genome-wide association studies identified eight genes that contribute to plasma levels of VWF in the normal population [4]. The *ABO* gene showed the strongest effect, but smaller effects were seen for the *STXBP5*, *SCARA5*, *VWF*, *STAB2*, *STX2*, *TC2N* and *CLEC4M* genes. Other studies have confirmed parts of the results above and identified an additional four genes [5, 6]. One linkage study identified a locus on chromosome 2 with an effect size on VWF variation comparable to the effect of the *ABO* locus. Detailed analysis identified a total of eight genes that may have an effect on VWF levels [7]. Additional studies of *STXBP5* and *STX2* [8] and *CLEC4M* [9, 10] have confirmed that single nucleotide variants (SNVs) in these genes are associated with the variation observed for plasma levels of VWF.

CLEC4M (C-type lectin member 4 family M) is a lectin receptor with a cytoplasmic domain, a transmembrane domain, a highly polymorphic neck region and a carbohydrate recognition domain. The carbohydrate recognition domain binds to molecules or cells that are glycosylated and this function is dependent upon the exact number of repeat units that are present in the neck region. The neck region consists of a 23 amino acid long motif that is repeated from three to nine times in different variants of CLEC4M. The neck region stabilizes CLEC4M by tetramerization of single CLEC4M molecules and affects the conformation of the receptor [11]. Previously genetic variation in the neck region of CLEC4M has been associated, for example, with susceptibility to infection by HIV [12] and SARS [13]. CLEC4M also binds to VWF [9] and variants in this gene contribute to the variation in the VWF level observed both in normal individuals [4] and in type 1 VWD patients [9, 10]. CLEC4M is coded for by the *CLEC4M* gene that is located on chromosome 19 and has seven exons located over 6.4 kbp. Exons 1–3 code for the cytoplasmic and transmembrane regions, exon 4 for the polymorphic neck region and exons 5–7 for the ligand binding domain of the CLEC4M protein. Exons 1 and 7 have untranslated regions and all exons have less than 200 bp of coding sequence, except for exon 4 which codes for the neck region and is the largest exon. Since the neck region consists of a 69-bp repeated sequence that is repeated from three to nine times, this region is difficult to sequence by conventional Sanger sequencing and is instead commonly analyzed by determining the number of repeat units [14]. Genetic diversity of the *CLEC4M* gene has been studied extensively by genotyping and re-sequencing in African and non-African populations [11, 15, 16].

The present study aimed to screen comprehensively for genetic variation in the *CLEC4M* gene in individuals from 106 unrelated type 1 VWD families by re-sequencing the gene region (excluding exon 4) and genotyping the polymorphic neck region (exon 4) of the gene. A first functional assessment of all variants was made using frequency comparisons between patients and controls and comparison with sequence data of the 1000Genomes project. Contrary to previous studies that have used tagSNPs to investigate the effects of *CLEC4M* variation on VWF level, the present study also screened for rare variants. The overall goal of the study was to investigate whether genetic variants in *CLEC4M* are associated with type 1 VWD in the Swedish population looking at both common and rare variants.

## Materials and methods

### Study populations and VWD phenotyping

The type 1 VWD study population was recruited at the Department for Coagulation Disorders, Malmö University Hospital (Malmö, Sweden). The population consisted of consecutive patients and their relatives who attended the clinic between the years 1988–2005 and corresponded to approximately 1000 individuals belonging to 126 families. This population represented the majority of all families diagnosed with type 1 VWD in Sweden during this time period. Clinical and laboratory data were recorded for each patient and their bleeding phenotypes were classified [17]. We used historical VWF levels usually determined at the time of the original diagnostic work-up. There were no further analyses of VWF levels in this study. Therefore, different phenotypical methods were used. VWF activity was measured with the traditional VWF:RCo method based on aggregation of platelets or an automated VWF:RCo assay based on the BCS coagulation analyzer using the BC von Willebrand reagent (Dade Behring Inc., Newark, DE, USA). VWF antigen levels (VWF:Ag) were measured with Electroimmunoassay (the Laurell method) and IRMA, ELISA, or LIA. VWF levels are given as IU/dl and were VWF:Ag median 39, min 10, max 63. The corresponding VWF activity values were VWF:RCo median 44, min < 5, max 71 (Table 1). Patients were characterized with respect to their VWF mutations in Manderstedt *et al*. [18]. Of these 126 families mutation analysis identified 20 families with type 2 mutations. The remaining 106 families were analyzed in the present study. In a strict sense, not all index cases fulfilled the modern definition of type 1 VWD, but at the time of diagnosis their bleeding symptoms in combination with lowered VWF levels were interpreted as reflecting type 1 VWD. More than two-thirds of the patients had a family history of bleeding, the remaining were individual patients with low VWF levels. Since the present study investigated one of the additional factors associated with VWF level variation, individuals with bleeding symptoms and low VWF levels were included in the study regardless of whether they had *bona fide VWF* mutations or not. In addition, two Swedish control populations were also analyzed: control population 1 (C_1_) consisting of 225 unrelated individuals from the general population and control population 2 (C_2_) consisting of 211 unrelated individuals from the general population with no history of bleeding [19]. These controls were recruited from the same geographical region as the patients included in the study. The present study was approved by the Ethics Committee of the Medical Faculty, Lund University, and the Swedish Data Inspection Board according to LU 436–01. Written informed consent was obtained from all subjects. DNA from human whole blood was isolated using a Qiagen Blood DNA kit (Qiagen, Hilden, Germany) and DNA concentrations were determined by fluorometry using PicoGreen® (Molecular Probes, Eugene, OR, USA).

**Table 1:** Baseline characteristics.

Characteristics	VWD1^a^
Age (years), median (range)	42 (1–74)
Child, n (%)	10 (9%)
Male sex, n (%)	31 (29%)
Blood group O, n (%)	75 (71%)
VWF:Ag (IU/dL), median (IQR)	39 (30–45)

^a^VWD1, the Swedish type 1 VWD population (n = 106); IQR, interquartile range

### Primer design

The Ensembl Genome Browser (http://www.ensembl.org) referring to the GRCh37.p13 version of the NCBI database contributed all DNA sequences that were used for primer design. Tandem Repeats Finder software was used to identify repeated sequences in the *CLEC4M* gene region (http://tandem.bu.edu/trf/trf.html) and masking of repeated sequences used the Ensembl Genome Browser. Primers were designed using Primer-BLAST (http://www.ncbi.nlm.nih.gov/tools/primer-blast/) and purchased from DNA Technology A/S (Risskov, Denmark).

### VNTR genotyping

The VNTR polymorphism in exon 4 of the *CLEC4M* gene was genotyped using the following PCR protocol: one cycle at 94°C for 3 min, followed by 10 cycles of 94°C for 15 s, 68°C for 30 s and 68°C for 1 min, then followed by 30 cycles at 94°C for 15 s, 67°C for 30 s and 68°C for 1 min, before a final extension cycle at 68°C for 10 min. The PCR reactions contained: 0.125 mM KAPA Taq DNA polymerase (KAPA Biosystems, Cape Town, South Africa), 1X KAPA Taq Extra Buffer, 1.75 mM MgCl_2_, 0.3 mM deoxyribonucleotide triphosphates, 2 ng/μl template DNA and 0.4 mM of each primer (S1 Table). The PCR products were visualized with gel electrophoresis on 30 x 40 cm 3% agarose gels (Seakem LE, Lonza, Rockland, ME, USA) using separation for 2 h at 10 V/cm. The genotyping results were validated in several ways: 1) The proper inheritance of alleles was confirmed by the analysis of three sets of trios (mother, father and child), 2) control populations C_1_ and C_2_ were used to confirm that the overall allele frequencies were as expected for background populations compared with previous studies, and 3) the type 1 VWD population was analyzed twice and evaluated independently by two persons. A few uncertainties were resolved by repeated analysis.

### DNA sequencing

Long range PCR (LRPCR) was used to amplify one amplicon including the *CLEC4M* promoter, exons 1–3 and intron 3 (promoter region) and another amplicon including intron 4 and exons 5–7 (the neck region and the ligand binding domain, S1 Table). The reactions used KAPA Taq Extra HS PCR Kit (KAPA Biosystems) as recommended by the manufacturer using the following PCR conditions: one cycle at 94°C for 3 min, followed by 40 cycles of 94°C for 15 s, 63°C for 30 s, 68°C for 4 min, and finally one cycle at 68°C for 10 min. The LRPCR products were then used as substrates in DNA sequencing reactions using a set of overlapping sequencing primers (S1 Table). Big Dye Terminator Sanger sequencing was performed in both directions using a 3130XL Genetic Analyzer (Applied Biosystems). Primary PCR products were treated with ExoSAP-IT® (Applied Biosystems) according to the manufacturer’s instructions and DNA sequencing was performed subsequently in a total volume of 5 μl containing 0.5X Big Dye sequencing ready reaction premix (Big Dye Terminator v 2.0, Applied Biosystems), 0.5X Big Dye Sequencing buffer and 3.2 pmol of the sequencing primer. The following PCR conditions were used: one cycle of 96°C for 1 min, 25 cycles of 96°C for 10 s, 50°C for 5 s and 60°C for 4 min. The sequencing reactions were purified using Xterminator (Applied Biosystems) according to the manufacturer’s instructions. Sequences were interpreted and all variants were identified using SeqScape ver. 2.5 and confirmed by manual inspection.

### SNP genotyping

Both the type 1 VWD and the C_1_ and C_2_ control populations were genotyped for rs868875, rs2277998, rs8113469 and rs62128260 using TaqMan-assays. The reactions were set up according to the manufacturer’s protocol and the genotyping was performed on a Bio-Rad CFX 384 with genotypes determined using CFX Manager™ Software (Bio-Rad Laboratories Inc., Hercules, CA, USA).

### Genetic analysis

Identified variants in the *CLEC4M* gene were compared to publicly available data in dbSNP (http://www.ncbi.nlm.nih.gov/SNP/) and 1000Genomes databases (http://www.1000genomes.org/). The 1000Genomes database consisted of 26 populations from different geographic regions. We extracted information from three of these populations: Utah residents of European descent (CEU, 99 individuals), Britons (GBR, 88 individuals) and Toscani residents (TSI, 107 individuals). The variants were obtained from the Integrated Variant Set, release April 2012, of the 1000Genomes Project available at http://ftp.1000genomes.ebi.ac.uk/vol1/ftp/release/20110521/. Variants were tested for Hardy-Weinberg equilibrium in the type 1 VWD patients. Single nucleotide variants were tested for association with disease status with a χ^2^ test as implemented in PLINK [20] using the 106 type 1 VWD index cases and the C_1_ and C_2_ individuals as controls. Haplotypes, haplotype blocks and linkage disequilibrium (LD) plots were constructed using Haploview 4.2 [21]. The functional consequences of missense variants were evaluated using SIFT [22], PolyPhen-2 [23] and MutationTaster [24].

## Results

### Screening for *CLEC4M* variants in the type 1 VWD population

The promoter and all exons and introns of *CLEC4M* except exon 4 were screened for variants in 106 individuals from unrelated type 1 VWD families by Sanger sequencing. The sequence data was generally of high quality with > 95% of bases having a Phred score of 30 or higher in > 95% of individuals. A total of 42 variants were found, 10 in the promoter, four in exons and 28 in introns of the gene (Table 2). Of the 42 variants, 37 were present in dbSNP and five were not. The previously unreported variants were all rare (minor allele frequencies [MAF] < 0.01), with three out of five variants detected in a single chromosome. A total of 10 variants had MAFs < 0.01 and 32 had MAFs ≥ 0.01. All SNPs were in Hardy-Weinberg equilibrium.

**Table 2:** *CLEC4M* variants in Swedish type 1 VWD patients (n = 106) and controls (n = 436) and in three 1000Genomes populations (n = 394).

SNP ID	Position^a^	Location	Allele^b^	VWD1^c^	C_tot_^c^	1000Genomes			
						Total	CEU^c^	GBR^c^	TSI^c^
rs11260029	7827576	5´Upstream	T/C	0.350	nd	0.279	0.293	0.250	0.290
NEW	7827590	"	A/G	0.009	nd	-	-	-	-
rs71581951	7827600	"	A/T	0.005	nd	0.002	0.005	-	-
rs149334204	7827639	"	T/C	-	nd	0.003	0.005	0.006	-
rs76634109	7827739	"	G/A	0.042	nd	0.063	0.071	0.051	0.065
rs76158574	7827742	"	T/G	0.005	nd	0.007	0.010	0.011	-
rs143606473	7827802	"	C/T	-	nd	0.003	0.005	-	0.005
rs183945146	7827803	"	G/A	0.005	nd	0.005	0.010	0.006	-
rs571497	7827830	"	G/A	0.117	nd	0.163	0.152	0.131	0.201
rs2287887	7827955	"	A/C	0.238	nd	0.284	0.268	0.295	0.290
NEW	7827969	"	T/A	0.009	nd	-	-	-	-
rs190409258	7828020	"	T/C	0.005	nd	-	-	-	-
rs117119495	7828177	Exon 1	G/A	-	nd	0.003	0.005	-	0.005
rs62623420	7828277	Intron 1	A/G	0.005	nd	0.009	0.010	0.011	0.005
rs186967614	7828542	Intron 2	G/A	-	nd	0.002	-	0.006	-
rs12977324	7829129	"	T/C	0.355	nd	0.277	0.283	0.250	0.294
rs113029049	7829141	"	G/A	-	nd	0.002	-	-	0.005
rs62126658	7829156	"	T/C	0.107	nd	0.129	0.121	0.080	0.178
rs538442	7829164	"	C/T	0.126	nd	0.149	0.136	0.210	0.107
rs12979429	7829180	"	G/A	0.346	nd	0.275	0.288	0.244	0.290
rs2335527	7829388	"	A/T	0.266	nd	0.313	0.288	0.301	0.346
rs582173	7829426	"	G/A	0.121	nd	0.129	0.116	0.142	0.131
rs2335528	7829539	"	T/G	0.271	nd	0.316	0.293	0.301	0.350
rs12986314	7829680	"	C/A	0.262	nd	0.311	0.293	0.295	0.341
rs189191378	7829812	"	C/T	-	nd	0.002	-	0.006	-
rs55731794	7829891	"	G/A	0.393	nd	0.414	0.429	0.455	0.364
rs594793	7829946	"	G/T	0.477	nd	0.419	0.414	0.358	0.477
rs868875	7831166	Intron 4	A/G	0.374	0.316	0.291	0.293	0.278	0.299
rs868876	7831226	"	A/T	0.355	nd	0.289	0.293	0.278	0.294
rs475896	7831429	"	G/C	0.486	nd	0.419	0.414	0.364	0.472
rs2277998	7831628	Exon 5	G/A	0.364	0.315	0.292	0.293	0.290	0.294
rs562607	7831700	Intron 5	C/G	0.150	nd	0.164	0.157	0.188	0.150
rs560634	7831953	"	G/T	0.140	nd	0.129	0.126	0.080	0.173
rs874492	7832001	"	A/T	0.383	nd	0.311	0.308	0.307	0.318
NEW	7832053	"	A/T	0.005	nd	-	-	-	-
rs558705	7832183	"	G/A	0.107	nd	0.116	0.096	0.114	0.136
rs76483925	7832218	"	G/C	-	nd	0.002	0.005	-	-
rs557094	7832286	"	C/G	0.150	nd	0.129	0.121	0.080	0.178
rs149388152	7832338	"	C/T	-	nd	0.002	-	-	0.005
rs2161525	7832664	Intron 6	T/C	0.322	nd	0.400	0.419	0.443	0.346
NEW	7832778	"	G/C	0.005	nd	-	-	-	-
rs655569	7832860	"	C/T	-	nd	0.002	0.005	-	-
rs8113469	7832959	"	T/C	0.234	0.271	0.270	0.242	0.227	0.332
rs12610506	7833071	"	G/A	0.430	nd	0.328	0.343	0.318	0.322
rs8105492	7833213	"	T/G	0.294	nd	0.273	0.247	0.222	0.341
rs148128064	7833232	"	C/T	-	nd	0.002	-	0.006	-
rs657855	7833408	"	C/T	0.136	nd	0.132	0.116	0.080	0.192
rs9329374	7833456	"	C/T	0.299	nd	0.268	0.242	0.222	0.332
rs141958280	7833653	"	A/G	-	nd	0.002	-	-	0.005
rs3745376	7833690	"	G/T	0.126	nd	0.158	0.177	0.176	0.126
rs148569581	7833820	"	C/T	-	nd	0.002	-	0.006	-
rs62128260	7833876	3´UTR	T/A	0.150	0.119	0.132	0.116	0.080	0.192
NEW	7833916	"	A/T	0.005	nd	-	-	-	-
rs67057658	7833940	"	G/A	0.126	nd	0.158	0.177	0.176	0.126

^a^In reference to GRCh37.p13, chromosome 19.

^b^Presented as major/minor allele.

^c^VWD1, Swedish type 1 VWD patient population (n = 106); C_tot_, two Swedish control populations (n = 436); CEU (n = 99), GBR (n = 88) and TSI (n = 107), three European populations from the 1000Genomes project.

nd, data not available. -, variant not found in the population.

**Fig 1:**
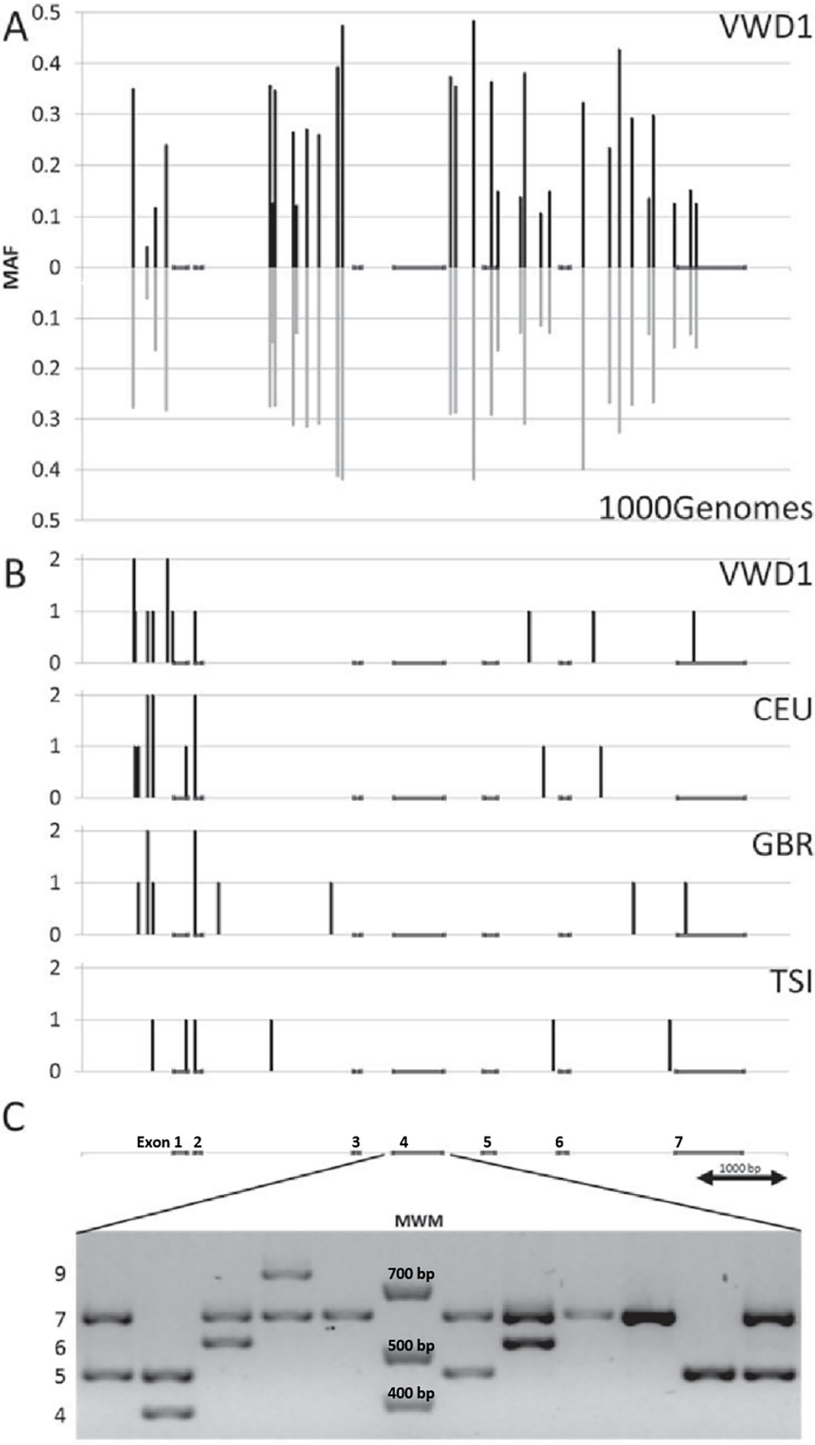
Common and rare variants in *CLEC4M*. (A) The positions and minor allele frequencies for common variants in *CLEC4M* in type 1 VWD patients (n = 106), and three 1000Genomes populations; CEU (n = 99), GBR (n = 88) and TSI (n = 107). (B) The positions and numbers of rare variants in the respective populations. (C) VNTR genotypes showing all common alleles.

Comparison of the results of the type 1 VWD population (106 individuals) with data from the similarly sized 1000Genomes populations CEU, GBR, and TSI (99, 88 and 107 individuals, respectively) showed that both the total number of variants (42 versus 41, 40 and 38) and the number of variants present in only one or two chromosomes (10 versus 8, 8, and six) were similar for the data sets both in number and in their distribution along the gene (Table 2, S2 Table and Fig 1). A total of 36 variants were common to the type 1 VWD and one or more of the 1000Genomes populations. These had in general high MAFs: 31 SNPs > 0.10, one SNP 0.01–0.10 and four SNP < 0.01. Pairwise comparisons between the type 1 VWD population and the three 1000Genomes populations identified similar number of variants unique to each population in all combinations. All of them were rare variants. Thus, no significant accumulation of rare variants was detected in the type 1 VWD population.

In the first part of the gene (promoter-intron 3) five SNPs had allele frequencies differing more than 5% between the type 1 VWD population and the sum of the 1000Genomes populations, but the allele frequencies of these five SNPs also varied considerably between the three 1000Genomes populations. In contrast, the ligand binding domain of the gene (intron 4-exon 7) contained seven SNPs with allele differences above 5%. For rs868875, rs868876, rs2277998 rs874492 and rs12610506 the observed differences between the type 1 VWD and the 1000Genomes populations were greater than two times the maximum internal difference observed for the three 1000Genomes populations. There were two variation deserts in the *CLEC4M* gene, one encompassing the first half of intron 2 and one covering intron 3 (Table 2 and Fig 1). The only SNP in the coding sequence of this gene was rs2277998, a G>A (Asp > Asn) missense mutation. The A-allele had a higher frequency in type 1 VWD compared with the 1000Genomes populations (0.364 versus 0.292). This missense mutation was predicted to be a tolerated variant by the three functional prediction programs: SIFT, PolyPhen and MutationTaster (S3 Table).

The ligand binding domain of the gene was analyzed further to ascertain whether the observed allele frequency differences were truly associated with type 1 VWD or if they were a result of variation due to ethnicity. Haplotyping using 1000Genomes data revealed rather strong LD across the entire *CLEC4M* gene region and allowed the selection of four tagSNPs: rs868875, rs2277998, rs8113469 and rs62128260. TaqMan genotyping was performed in the two Swedish control populations (C_1_ and C_2_) and in the type 1 VWD population. The allele frequencies of all four SNPs were similar in the C_1_, C_2_ and 1000Genomes populations. All four SNPs showed allele frequency differences between the type 1 VWD and the control populations. The largest frequency difference and the lowest *P*-value was observed for rs868875 (5.8%, *P* = 0.10). The remaining SNPs showed a gradual decrease in their frequency differences with increasing distance from the rs868875 SNP (Table 2).

### Genotyping the VNTR in *CLEC4M*

The type 1 VWD population and the C_1_ and C_2_ control populations were genotyped for the VNTR in exon 4 of *CLEC4M*. The alleles with five, six and seven repeat units were the most frequent and together explained > 94% of all alleles. Only small allele frequency differences were observed between the two control populations, whereas the type 1 VWD population showed an increase of allele 5 (3.2%) and a decrease of allele 7 (3.0%) compared with the control populations (Table 3 and Fig 1).

**Table 3:** *CLEC4M* VNTR allele frequencies in Swedish type 1 VWD patients and controls.

Allele	Controls				VWD1	
	C_1_	C_2_	C_tot_	C_1_-C_2_	VWD1	VWD1-C_tot_
4	2.6	3.1	2.8	-0.5	4.2	1.4
5	28.8	29.4	29.1	-0.6	32.2	3.2
6	12.8	12.7	12.8	0.1	12.6	-0.1
7	52.2	52.9	52.5	-0.7	49.5	-3.0
8	0.2	0.0	0.1	0.2	0.0	-0.1
9	3.5	1.8	2.7	1.7	1.4	-1.3

C_1_+C_2_ = C_tot_, two Swedish control populations (n = 225+211 = 436); VWD1, type 1 VWD patient population (n = 106).

The allele frequencies observed for each population were used to calculate the expected genotype frequencies and these were subsequently compared with the observed genotype frequencies. With seven different possible alleles (alleles 3–9), corresponding to a total of 28 different possible genotypes, this could potentially result in a very complex table. As a result of alleles 5, 6 and 7 in all possible pairwise combinations together explaining 89% of all genotypes, Table 4 only shows the results for these six genotypes. The differences between the expected and observed genotype frequencies within each control population were generally small. The average deviation was 1.0%, with a maximum deviation of 2.6% for genotype 67 in C_2_. Also, the observed differences between the C_1_ and C_2_ populations were small, with an average deviation of 1.0% and a maximum value of 1.4%. Contrary to this pattern, the type 1 VWD population showed three larger differences between the observed and expected genotype frequencies: surpluses of genotypes 57 (6.4%; *P* = 0.27) and 67 (6.2%; *P* = 0.18) and a deficiency of genotype 77 (5.8%; *P* = 0.31). The average deviation for the remaining genotypes was 2.4%. Comparing the observed genotype frequencies of the type 1 VWD population with the average of the C_1_ and C_2_ populations resulted in surpluses of genotypes 57 (7.4%; *P* = 0.13) and 67 (3.1%; *P* = 0.41) and a deficiency of genotype 77 (8.4%; *P* = 0.08) (Table 4). Thus, the pattern of deviating genotype frequencies observed within the type 1 VWD population corresponds to the pattern observed for the comparison of the type 1 VWD population versus the control populations.

**Table 4:** Expected and observed genotype frequencies of the *CLEC4M* VNTR.

Geno-type	C_1_			C_2_			C_1_-C_2_	VWD1			
	E	O	O-E	E	O	O-E	O-O	E	O	O-E	O-C_tot_
55	8.3	7.8	-0.5	8.7	9.0	0.4	-1.2	10.4	7.5	-2.9	-0.9
56	7.4	5.6	-1.7	7.5	6.7	-0.8	-1.0	8.1	4.7	-3.5	-1.5
57	30.0	31.2	1.1	31.1	30.6	-0.6	0.6	31.9	38.3	6.4	7.4
66	1.6	1.3	-0.3	1.6	0.8	-0.8	0.5	1.6	0.9	-0.7	-0.1
67	13.3	15.2	1.8	13.5	16.1	2.6	-0.9	12.5	18.7	6.2	3.1
77	27.2	26.4	-0.8	28.0	27.8	-0.2	-1.4	24.5	18.7	-5.8	-8.4

E, Expected genotype frequencies; O, Observed genotype frequencies; C_1_+C_2_ = C_tot_, two Swedish control populations (n = 225+211 = 436); VWD1, type 1 VWD population (n = 106).

### *CLEC4M* haplotyping

The type 1 VWD, C_1_ and C_2_ populations were genotyped using rs868875, rs2277998, rs8113469 and rs62128260 and the VNTR locus and the genotype data were used to construct combined SNP and VNTR haplotypes for all three populations. Two single haplotypes, one containing VNTR allele 3 and one with allele 8, were excluded. The remainder consisted of 19 haplotypes and six of these showed frequencies > 1% in the type 1 VWD population (Table 5). A number of observations can be made: 1) Each VNTR allele was primarily associated with one specific SNP haplotype. The only exception to this pattern was VNTR allele 7 that was associated with two relatively common SNP haplotypes. 2) All haplotypes with VNTR allele 4 had the same SNP haplotype (GATT). This haplotype was also the dominating haplotype for VNTR allele 5. 3) All haplotypes with VNTR allele 9 (with one exception) had the same SNP haplotype (AGCT). This haplotype was also one of the two dominating haplotypes for VNTR allele 7. 4) All SNP-VNTR haplotypes present at frequencies > 1% were present in both the type 1 VWD population and the control populations at similar frequencies. 5) The major frequency differences between the type 1 VWD population and control populations were seen for the 5GATT (surplus of 3.5% in type 1 VWD population) and for the 7AGTT haplotype (deficiency of 3.4% in type 1 VWD population).

**Table 5:** Combined SNP-VNTR haplotype frequencies.

Haplo-type	4		5		6		7		9		SNP^a^	
	VWD1	C_tot_	VWD1	C_tot_	VWD1	C_tot_	VWD1	C_tot_	VWD1	C_tot_	VWD1	C_tot_
AGTT	-	-	-	-	0.9	0.9	36.9	40.3	-	0.1	37.9	41.4
GATT	4.2	2.8	30.8	27.3	-	-	-	-	-	-	35.0	30.1
AGCA	-	-	-	-	10.7	11.0	0.9	0.4	-	-	11.7	11.5
AGCT	-	-	-	-	-	0.7	10.3	11.5	2.3	2.7	12.6	14.8
GGTT	-	-	0.9	0.2	0.9	0.1	-	0.2	-	-	1.9	0.6
AATT	-	-	-	-	-	-	0.9	0.8	-	-	0.9	0.8
AGTA	-	-	-	-	-	-	0.5	-	-	-	0.5	-
GACA	-	-	-	0.2	-	-	-	-	-	-	0.0	0.2
GACT	-	-	-	0.1	-	-	-	-	-	-	0.0	0.1
GATA	-	-	0.5	0.4	-	-	-	-	-	-	0.5	0.4
GGCT	-	-	-	0.1	-	-	-	-	-	-	-	0.1
VNTR^b^	4.2	2.8	32.2	28.4	12.6	12.7	49.5	53.2	2.3	2.8		

VWD1, type 1 VWD patients (n = 106); Ctot, Swedish control population (n = 436).

^a^Sum of SNP haplotype frequencies.

^b^Sum of VNTR allele frequencies.

## Discussion

Genetic variation in *CLEC4M* has been associated with variation in plasma levels of VWF in healthy individuals [4]. It has also been shown that the CLEC4M protein binds to and internalizes VWF, and variants in *CLEC4M* contribute to the variability of VWF plasma levels in type 1 VWD patients [9, 10]. Family-based association analysis of 318 type 1 VWD patients and 173 unaffected family members showed excess transmission of VNTR allele 6 to unaffected individuals and an association of this allele with increased VWF:RCo [9]. The rs868875 variant in *CLEC4M* showed association with both VWF level and activity in 364 type 1 VWD patients from the Netherlands [10]. Thus, CLEC4M was suggested to be causally involved in the development of type 1 VWD. The authors suggested a mechanism where certain variants of this protein are more efficient in clearing VWF from the circulation than others. An explanation model where the protein is more efficient in clearing VWF from the circulation may mean that the *CLEC4M* gene exists in a number of forms with differences in their level of expression or with differences in their affinity for VWF. Such differences may depend upon common mutations that are present for example in the promoter, the 3´UTR, the neck region or the ligand binding domain of the protein. That is, certain haplotypes are expected to be more common in patients compared with controls if common variants are operating to cause type 1 VWD. If rare variants contribute to type 1 VWD, an accumulation of rare variants in patients relative to controls is expected.

The present study analyzed a historical VWD population. This may have introduced a bias compared with the analysis of VWD populations using contemporary definitions. A recent study in the United States investigated 482 patients historically diagnosed with type 1 VWD [25]. When these patients were retested 172 patients did not meet the current diagnostic criteria for type 1 VWD or low VWF level (VWF:Ag < 50 IU/dl or VWF:RCo < 53 IU/dl). There was also no difference in bleeding score whether or not the current criteria were fulfilled. Complete VWF resequencing showed that 45% of the 482 patients with historical type 1 VWD diagnosis carried a rare variant in the VWF gene compared to 62% of the 310 patients fulfilling the modern criteria. Thus, this type 1 VWD population is very similar to our type 1 VWD population [18].

### Rare and common variants

The five new variants detected in the promoter, introns and 3´UTR were all rare (MAF < 0.01), with three out of five variants detected in a single chromosome. They could therefore potentially explain only a very minor fraction of the occurrence of the disease, since the rare new variants have no obvious functional consequences and were all present in patients already carrying *bona fide VWF* mutations [18].

However, several earlier reports associate the common SNV rs868875 with low VWF levels in healthy subjects and type 1 VWD patients. Similarly to these reports, the MAF of rs868875 was increased in the present study population (5.8%; *P* = 0.10) though the increase failed to reach significance. Except for rs2277998, no missense or nonsense mutations were detected. This particular variant was also present at a higher frequency in the type 1 VWD population compared with the Swedish control populations (0.364 versus 0.315; *P* = 0.21). Assuming that this amino acid change may somehow alter the function of the ligand binding domain of the CLEC4M protein, resulting in a higher affinity for and more efficient binding of VWF molecules in the bloodstream, the increased allele frequency in the type 1 VWD population relative to the control populations (4.9%) was compatible with an explanation model where rs2277998 contributes to type 1 VWD.

### VNTR variants

When the VNTR allele and genotype frequencies were compared in two different Swedish control populations only small frequency differences were observed, whereas the type 1 VWD population showed larger differences compared with the control populations for some alleles and genotypes; e.g. genotypes 57 (7.4%) and 77 (-8.4%). In addition, when the allele frequencies were used to calculate the expected genotype frequencies and these were then compared with the observed frequencies only small frequency differences were observed in the two control populations. Again, the type 1 VWD population showed several distinct genotype frequency differences for genotypes involving the alleles 5 and 7, namely genotypes 57, 67 and 77 at frequency differences of 6.4%, 6.2% and -5.8%, respectively. Thus, the frequency differences were increased when going from alleles to genotypes. In addition, both heterozygous genotypes were observed at higher frequencies than expected, whereas the homozygous genotype was observed at a lower frequency than expected. This strongly indicates an association between VNTR heterozygosity and type 1 VWD.

### Identifying disease determinants

Earlier reports have reported associations between rs868875 and type 1 VWD [9, 10] and between VNTR allele 6 and type 1 VWD [9]. Strong LD between rs868875, rs2277998 and the VNTR locus complicates determining whether either or all of them contribute to type 1 VWD. From a naïve functional analysis, there is no obvious explanation indicating an effect of rs868875 since it is intronic, but both rs2277998 and the VNTR might affect the affinity for VWF. Previous biochemical studies have shown large effects on tetramerization and stability of tetramers due to the different sizes of VNTR alleles. VNTR alleles with six or more repeats readily tetramerize whereas allele 5 gives rise to equilibrium between monomers and tetramers. Alleles of length four do not tetramerize at all [26]. Other studies have shown differences in *in vitro* binding affinity due to differing neck length both with regard to carbohydrates [27] and to VWF itself [9]. Several reports also link neck length variation to variation in infection rates for viruses such as HIV [12] and SARS-CoV [13] both on the allelic and genotypic levels; other reports indicate that the HIV transfection efficiency is due to the number of CLEC4M proteins on the cell surface [28]. Specifically, the number of proteins on the cell surface depends on combinations of VNTR (alleles 5, 7, 9) and SNP (rs2277998; A, G) alleles. They showed that constructs containing 5A and 7G gives rise to the highest numbers of proteins on the cell surface. These combinations are in almost complete linkage disequilibrium in both cases and controls in the present study. The largest enrichment between patients and controls found in the present study is the higher frequency of the 57 VNTR genotype (7.4%). In addition, this genotype is more increased than what would be expected from the increase in VNTR allele frequencies alone (6.4%). Unfortunately, the study by Zhu et al. [28] did not include heterozygote combinations of these two alleles compared with the respective homozygotes. Also, the strong LD makes it impossible to differentiate the effects of the VNTR and the rs2277998 SNP and there is a lack of studies of the functional effect of the Asp > Asn substitution caused by rs2277998 with regards to VWF. But since this missense variant was predicted to be tolerated by the three functional prediction programs SIFT, PolyPhen and MutationTaster it seems to be a less likely candidate than the VNTR genotypes.

Earlier work involving CLEC4M and its relation to infectious disease has generated much debate regarding reported associations [13, 29, 30]. The analysis is complicated by the fact that the ligand binding domain shows large population-specific variation and seems to be under balancing selection [15], which makes any analysis vulnerable to undetected population stratification and confounding factors such as selection driven by different microbial interactions. In this study two control populations were used to investigate whether there is any population stratification. Both populations were collected from the same geographical area as the type 1 VWD population. The genetic variation in the two control populations was very similar. In contrast to the type 1 VWD population they showed no skewness with regards to the VNTR genotypes, while the type 1 VWD population was highly skewed in favour of heterozygotes. Nonetheless, the rather small effect size and the multi-allelic structure of the VNTR require larger studies or a meta-analysis to determine the impact of the VNTR polymorphisms on type 1 VWD.

Previous studies have shown that VWF levels of individuals with blood group O are reduced by 30% in comparison to non-O individuals and that blood group O is overrepresented in type 1 VWD patients [2]. In the present study 75 index cases (71% of 106 type 1 VWD index cases) were of type O, clearly higher than the Swedish national average of ~ 40% type O. Compatible with other studies, no overrepresentation of type O was found in index cases carrying a type 2 VWD mutation (25%). Thus, the patients analyzed had a higher incidence of blood group O as would be expected. This verifies that the patients being analyzed in the present study are enriched for factors other than *bona fide VWF* mutations that affect the VWF levels. Since blood group O is by far the strongest of these factors the corresponding enrichment is obvious. The additional factors that have been identified and confirmed in a number of studies show considerably lower individual effects of ~ 5%. One of these other factors is genetic variation in *CLEC4M*. Our results are compatible with previous findings and confirm the role of certain VNTR genotypes and the common missense variant. The re-sequencing in the present study could not detect any obvious sign of an additional contribution from rare variants that individually could have a larger effect on the phenotype. However, smaller effects cannot be excluded due to the small population size. This means that the *CLEC4M* effect on the VWF level in the majority of cases is limited to the contribution from the common haplotype.

## Supporting information

S1 Table Primers for *CLEC4M*. (XLSX)

S2 Table Genetic variants found in *CLEC4M* per individual. (XLSX)

S3 Table MutationTaster predictions for SNVs found in *CLEC4M*. (XLSX)
